# Photocatalytic Cleanability of ZnO-Decorated Ceramic Membranes for Rhodamine B Removal

**DOI:** 10.3390/membranes16040148

**Published:** 2026-04-14

**Authors:** Yassine Khmiri, Feryelle Aouay, Afef Attia, Hajer Aloulou, Lasâad Dammak, Catia Algieri, Raja Ben Amar

**Affiliations:** 1 Research Unit “Advanced Technologies for Environment and Smart Cities”, Faculty of Sciences, University of Sfax, Sfax 3000, Tunisia; yasssinlac@gmail.com (Y.K.); feryel.aouay@gmail.com (F.A.); afef.attia@fss.usf.tn (A.A.); hajer.aloulou89@yahoo.fr (H.A.); 2 ICMPE UMR-CNRS 7182-UPEC, Université Paris-Est Créteil, 2 Rue Henri Dunant, 94320 Thiais, France; 3 Department of Chemistry, Preparatory Institute for Engineering Studies of Gabes, University of Gabes, Gabes 6029, Tunisia; 4 Institute on Membrane Technology “Enrico Drioli”, National Research Council of Italy (ITM–CNR), Cubo 17C, Via Pietro Bucci, 87036 Rende, Italy; c.algieri@itm.cnr.it

**Keywords:** photocatalytic membrane, RhB dye degradation, photocatalytic regeneration, ZnO deposition, spin coating

## Abstract

The widespread presence of stable and hazardous organic contaminants, such as synthetic dyes, in industrial effluents necessitates the development of resilient treatment strategies capable of achieving efficient degradation and decolorization of dye pollutants. Conventional treatment processes often fail to remove such recalcitrant compounds, prompting growing interest in integrated advanced systems. Photocatalytic membranes represent a promising solution due to the synergistic combination of physical separation and catalytic degradation. In this study, zinc oxide (ZnO) thin films were deposited by spin coating onto smectite–zeolite ceramic membranes (MS10/Z90), applying one (M1), two (M2), and three (M3) successive coating layers to control catalyst thickness. SEM analysis confirmed that increasing the number of layers resulted in a thicker and more homogeneous ZnO coating, while XRD revealed enhanced crystallinity and larger crystallite size. Water permeability decreased progressively from 623 L·h^−1^·m^−2^·bar^−1^ for the uncoated membrane to 506, 439, and 350 L·h^−1^·m^−2^·bar^−1^ for M1, M2, and M3, respectively. Photocatalytic performance was evaluated using Rhodamine B (RhB) (10 mg·L^−1^) under UV irradiation (365 nm, 18 W) for 180 min, achieving degradation efficiencies of 83.0%, 94.6%, and 99.1% for M1, M2, and M3, respectively. The degradation kinetics followed a pseudo-first-order model, with rate constants increasing with catalyst layer thickness. Free radical scavenging assays confirmed that hydroxyl radicals (•OH) were the primary reactive species responsible for RhB degradation. These findings highlight the critical influence of ZnO layer thickness and mass transfer on photocatalytic performance, demonstrating the potential of ZnO-coated ceramic membranes for efficient pollutant degradation and in situ photocatalytic regeneration. Permeability measurements after photocatalytic treatment confirmed effective flux recovery, supporting the operational durability of the developed membranes.

## 1. Introduction

Industrial activities remain one of the primary drivers of global water pollution, as industrial effluents contain complex mixtures of refractory organic pollutants [1], inorganic salts, heavy metals, and toxic compounds that are poorly removed by conventional treatment processes [2,3]. As one of the most water-intensive sectors [4,5], the textile industry generates a significant pollution burden. With the production of one ton of textiles requiring 100–200 m^3^ of water, of which 80–90% becomes wastewater [6], vast quantities of chemically complex and highly colored effluent are discharged into aquatic environments [1,7]. Among synthetic dyes, cationic dyes such as Rhodamine B (RhB) are of particular concern due to their high molecular stability, resistance to biodegradation, and strong affinity for aquatic systems [8]. RhB is widely used in the textile, rubber, and paper industries and is frequently detected in industrial effluents at concentrations ranging from 20 to 300 mg·L^−1^ [9]. This dye is associated with toxic, mutagenic, and potentially carcinogenic effects (Group 3 classification) and can disrupt aquatic ecosystems even at trace concentrations. Therefore, its efficient removal requires advanced treatment technologies capable of both separation and degradation [10].

Membrane-based separation processes have emerged as a key technology for dye removal owing to their high rejection efficiency, compact design, and operational flexibility [11,12]; However, their long-term performance is severely constrained by membrane fouling, which arises from the adsorption, deposition, and concentration polarization of pollutants on the membrane surface and within its pores [13]. This phenomenon leads to flux decline, increased transmembrane pressure, higher energy consumption, and reduced membrane lifespan, particularly in highly contaminated effluents [14,15].

In parallel, photocatalysis has been extensively investigated as a promising oxidation process for the degradation of organic pollutants through the generation of reactive oxygen species (ROS), including hydroxyl radicals (•OH), superoxide radicals (O_2_•^−^), and hydrogen peroxide (H_2_O_2_) under light irradiation [16]. Nevertheless, conventional slurry-based photocatalytic systems present major limitations, including catalyst agglomeration, nanoparticle loss, difficult post-treatment recovery, and reduced efficiency at high pollutant loads due to light scattering and limited photon penetration [17].

To overcome these challenges, the integration of membrane filtration with photocatalysis into a single hybrid system has gained significant attention [18]. Immobilizing photocatalytic materials onto a membrane surface enables simultaneous physical separation and in situ oxidative degradation of pollutants, preventing catalyst loss while promoting continuous cleanability of the membrane under light irradiation and improving permeability recovery [19]. Recent reviews have extensively discussed the rapid development of photocatalytic membrane reactors for wastewater treatment, highlighting their hybrid separation-oxidation functionality [19,20,21].

In this context, zinc oxide (ZnO) has emerged as a highly promising photocatalytic surface modifier owing to its favorable semiconductor properties, strong photoactivity, and chemical stability. Baig et al. [22] showed that ZnO thin films deposited on alumina membranes via RF magnetron sputtering form a uniform, stable coating with pronounced photo responsiveness, attributed to efficient photogenerated charge separation under light irradiation. Kuang et al. [23] further demonstrated that ZnO layers grown on Al_2_O_3_ supports enhance photocatalytic performance through effective light absorption and electron–hole pair formation while maintaining strong interfacial adhesion with the ceramic substrate. In addition, Wei et al. [24] reported that ZnO nanoflower architectures provide a high specific surface area and abundant active sites, significantly boosting photocatalytic reactivity when integrated onto ceramic membranes. Earlier investigations by Huang et al. [25] also confirmed that nanostructured ZnO coatings promote enhanced generation of reactive oxygen species (ROS) and improve surface redox activity, which are critical for efficient degradation of organic pollutants. Collectively, these studies indicate that the photocatalytic performance of ZnO-decorated ceramic membranes is strongly governed by ZnO morphology, coating uniformity, crystallinity, and interfacial stability with the membrane support. Despite these promising advances, most existing photocatalytic membrane systems still rely on solar irradiation or complex reactor configurations [26]. Recent reviews on catalytic membranes emphasize the need for simple and reproducible reactor designs for wastewater treatment [27]. Therefore, developing robust photocatalytic membrane systems operating under artificial light for highly colored textile wastewater remains a key challenge.

The novelty of the work lies in (i) systematically controlling ZnO thickness via successive spin-coated layers, (ii) correlating structural evolution with degradation kinetics, and (iii) experimentally validating cleanability capability through post-reaction structural analyses. This work aims to develop a novel ZnO-functionalized ceramic membrane that integrates size-selective filtration and photocatalytic regeneration within a single, robust platform for dye-contaminated wastewater treatment. The system is designed to enable in situ degradation of accumulated organic pollutants under UV irradiation, potentially reducing irreversible fouling and extending membrane operational lifetime. The membrane is rationally designed by coating a mesoporous ceramic support with immobilized nanostructured ZnO, forming a bifunctional interface capable of simultaneously retaining RhB and photocatalytically degrading it at the membrane–water interface under light irradiation.

Unlike conventional membranes that suffer from surface fouling, the proposed system directly degrades the accumulated organic layer in situ through reactive oxygen species generated by ZnO, thereby addressing fouling at its origin rather than through periodic external cleaning. In addition, this study introducesa fixed-bed, submerged multilayer ceramic photocatalytic membrane reactor that eliminates catalyst leaching, avoids slurry handling and post-recovery steps, and ensures reproducible photocatalyst activation using an external artificial light source. By combining separation and degradation in a single reusable membrane, this approach enables sustained permeate flux, reduced maintenance and energy demand, and extended membrane lifespan, offering a promising approach for improving photocatalytic membrane performance in dye-contaminated wastewater treatment.

## 2. Materials and Methodology

### 2.1. Chemicals

Zinc oxide (ZnO) (≥99% purity), Rhodamine B (≥95% purity), and polyvinyl alcohol (PVA (98.99)), isopropanol (IPA), ethylenediaminetetraacetic acid disodium salt (EDTA-2Na), and ascorbic acid (ASC), were employed in this study, all purchased from Sigma-Aldrich (St. Louis, MO, USA). Table 1 summarizes the key properties of the model dye used. The flat ceramic support (MS10/Z90), consisting of 10 wt% smectite and 90 wt% zeolite, has been described in detail in a previous study. The support has a diameter of 55 mm and a thickness of 3 mm [28] (Table 2).

### 2.2. Deposition of ZnOThin Film Active Layer on Support MS10/Z90 Using Spin Coating

**Figure 1:**
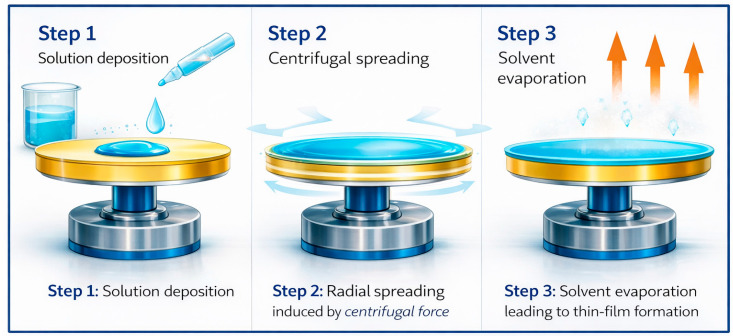
Centrifugal coating process for thin-film deposition: (1) solution deposition, (2) rotational spreading, (3) solvent evaporation.

The active layer was fabricated from ZnO powder using a spin-coating technique [26] applied to the MS10/Z90 substrate [28]. A coating solution was prepared by dispersing 4 g of ZnO particles in 100 mL of deionized water. Separately, the PVA solution was prepared by dissolving 2 wt% PVA in deionized water under magnetic stirring at 80 °C until complete dissolution. The ZnO dispersion and the PVA solution were then mixed in a 1:1 volume ratio, yielding a final ZnO concentration of 4 wt% in the coating suspension. The mixture was homogenized under magnetic stirring for 2 h to obtain a stable colloidal suspension. [29]. The coating process is illustrated in Figure 1.

The fabrication process began with careful preparation of the substrate. One side of the ceramic support was polished to ensure surface uniformity, followed by ultrasonic cleaning in deionized water to remove abrasive residues and contaminants. The substrate was then dried overnight in an oven at 105 °C to achieve a completely dry and thermally stable surface, providing an optimal foundation for the functional layer.

The coating suspension was synthesized by preparing two separate solutions: ZnO powder dispersed in water to form a suspension, and an aqueous PVA solution serving as the binding agent. Equal volumes of these solutions were blended and homogenized using ultrasonic energy, yielding a stable colloidal mixture. Multiple coating layers of this suspension were applied to fabricate membranes with varying ZnO loadings. The resulting lines deposited on the membrane surface are detailed in Table 3.

Deposition was performed using a centrifugal coating method. A precise volume of the colloidal suspension was dispensed onto the rotating substrate at a controlled flow rate. Spinning at 400 rpm ensured uniform spreading of the liquid across the surface, while centrifugal force expelled the excess, forming a consistent thin film.

The consolidation phase involved carefully controlled thermal treatment. The freshly coated membrane was first air-dried for 24 h to allow gradual solvent evaporation. It then underwent a two-stage heating process: binder removal at 300 °C to pyrolyze the organic PVA, followed by sintering at 500 °C to densify the ZnO structure [30]. This sintering temperature was chosen to enhance ZnO crystallinity and photocatalytic activity while preventing excessive grain growth and preserving membrane integrity.

### 2.3. Membrane Characterization

The membranes were characterized using Fourier Transform Infrared Spectroscopy (FTIR, Spectrum 100, PerkinElmer, Waltham, MA, USA) and X-ray Diffraction (XRD, D8 Advance diffractometer, Bruker, Billerica, MA, USA). XRD measurements were carried out with Cu K_α_ radiation (λ = 1.5406 Å) over a 2θ range of 4–80°. The surface morphology of the membranes was further examined using scanning electron microscopy (SEM, Hitachi S4800, Tokyo, Japan). Also, the chemical composition of the membrane samples was detected using energy-dispersive spectroscopy (EDX) and elemental mapping analysis.

Membrane porosity (ε) was determined according to Archimedes’ principle. The membrane sample was first dried at 100 °C for 6 h and weighed to obtain the dry mass (W_1_). It was then immersed in deionized water for 24 h to ensure complete saturation. After carefully removing excess surface water, the saturated membrane was weighed to determine the wet mass (W_2_). The porosity was subsequently calculated using Equation (1).

(1) $$\varepsilon =\frac{W_{1}-W_{2}}{\rho_{water}\times V_{mem}}\times 100$$

where W_1_ and W_2_ represent the dry and wet membrane weights, V_mem_ corresponds to the membrane volume, and ρ_water_ denotes the density of water.

### 2.4. Membrane Performance

Membrane permeability was assessed at a temperature of 25 °C and a transmembrane pressure (TMP) ranging between 1 and 3 bar using distilled water. Before experiments, the membrane was conditioned by immersion in distilled water for at least 24 h. The working pressure was kept constant using a nitrogen gas source, which was controlled by a control pressure gauge and a purge valve. The membrane permeability was evaluated in a dead-end filtration configuration by measuring the variation in distilled water flux J_w_ (L·h^−1^·m^−2^) with TMP (bar), in accordance with Darcy’s law (Equation (2)) [31]:

(2) $$J_{w}=L_{p}\times \Delta P$$

**Figure 2:**
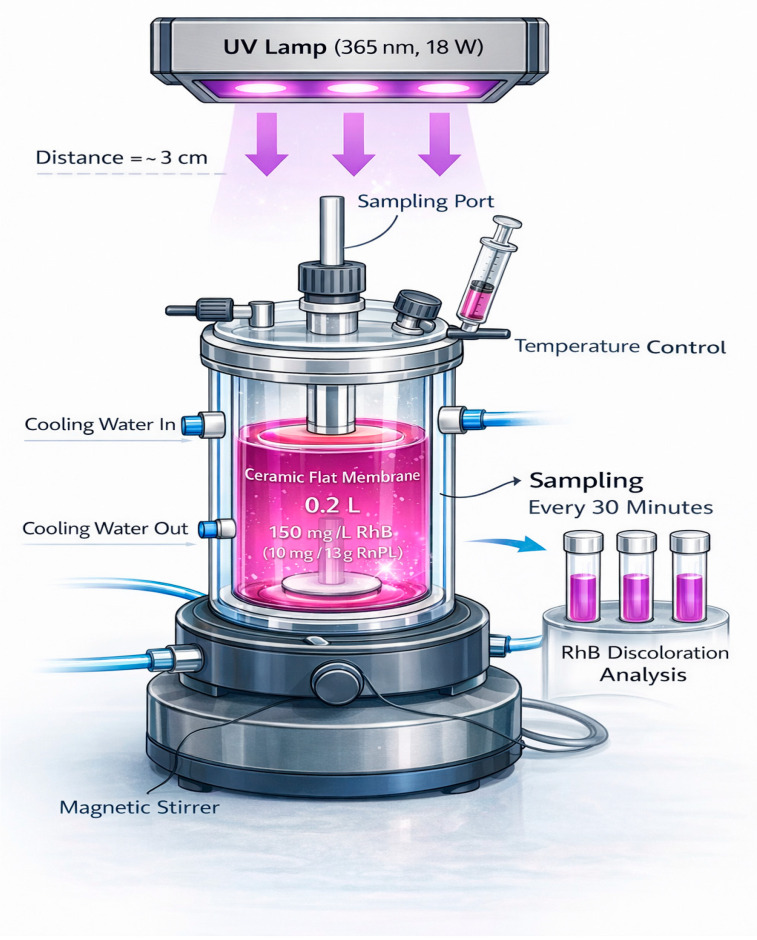
Photocatalytic reactor.

In this study, the photocatalytic activity of the samples was assessed by monitoring the degradation of RhB under UV irradiation. The schematic of the photoreactor is shown in Figure 2. The experiments were conducted in a double-jacketed glass reactor (total volume: 0.2 L) containing 150 mL of RhB solution at an initial concentration of 10 mg·L^−1^. The pH of the solution was maintained at 6.5 ± 0.2. This concentration was selected as it is widely used in photocatalytic studies, enabling comparison with previous results [32,33]. In addition, 10 mg·L^−1^ provides sufficient absorbance for accurate spectrophotometric analysis without significant mass transfer or light-screening effects.

A UV lamp (λ = 365 nm, 18 W) was positioned at a distance of 3 cm from the membrane surface, providing an illuminated area of 23.75 cm^2^. The irradiance at the membrane surface was measured to be 4.2 mW/cm^2^ using a radiometer. This distance was carefully selected to provide effective irradiation of the active layer while minimizing thermal effects and ensuring homogeneous light distribution across the membrane surface [34]. Aliquots of the solution were withdrawn every 30 min to evaluate the progress of RhB degradation. The decrease in RhB concentration was monitored by measuring the absorbance at 556 nm, corresponding to the maximum absorption wavelength of the chromophore. This decrease reflects the disappearance of the characteristic absorption band and is attributed to both decolorization and degradation of the RhB chromophore structure [26]. The photocatalytic degradation efficiency was calculated according to Equation (3).

(3) $$Degradation(\%)=\frac{\left(C_{0}-\left.C_{t}\right)\right.}{C_{0}}\times 100$$

where C_0_ is the initial RhB concentration (mg·L^−1^), and Ct is the RhB concentration at time t.

The calibration curve is used to calculate the RhB concentration (C) based on the Beer–Lambert law (Equation (4)):

(4) A=ε×l×C

where A is the absorbance, ℰ is the molar extinction coefficient (L·mol^−1^·cm^−1^), l is the optical path length (1 cm), and C is the dye concentration (mg·L^−1^).

### 2.5. Kinetics Study

The photocatalytic degradation kinetics of RhB in the aqueous phase were described using the Langmuir–Hinshelwood model (Equation (5)) [35]:

(5) $$r=\frac{dC}{dt}=\frac{-kk_{e}C}{1+k_{e}C}$$

where r is the reaction rate (mg·L^−1^·min^−1^), C is the pollutant concentration (mg·L^−1^), k is the Langmuir–Hinshelwood reaction rate constant (mg·L^−1^·min^−1^), and k_e_ is the Langmuir adsorption equilibrium constant (L·mg^−1^).

At low initial pollutant concentrations (kC<<1), the model simplifies to pseudo-first-order kinetics (Equation (6)):

(6) $$Ln(\frac{C_{0}}{C_{C}})={k_{app}}^{\prime }t$$

where k_app_ is the apparent rate constant (min^−1^), C_0_ is the initial RhB concentration (mg·L^−1^), C is the RhB concentration at time t (mg·L^−1^), and t is the reaction time (min). The apparent rate constant was determined from the slope of the linear plot of ln (C_0_/C) versus time.

### 2.6. Scavenger and Degradation Studies

Investigating the photodegradation pathway of RhB required the identification of the dominant reactive oxidants. Therefore, a series of scavenging experiments were performed using isopropanol (IPA), ethylene diamine tetra-acetic acid (EDTA-2Na), and ascorbic acid (ASC) to selectively inhibit hydroxyl radicals (•OH), photogenerated holes (h^+^), and superoxide radicals (O_2_•^−^), respectively, based on established protocols [36,37]. By comparing the degradation rates upon the addition of these quenchers, the contribution of each species to the overall conversion of RhB was assessed. For the degradation experiments, a scavenger concentration of 0.5 mmol·L^−1^ was used [38]. The extent of RhB removal was subsequently determined by collecting reaction aliquots (40 mL) and analyzing them via UV–vis spectroscopy.

## 3. Results and Discussion

### 3.1. Membrane Characterization

#### 3.1.1. XRD Analysis

**Figure 3:**
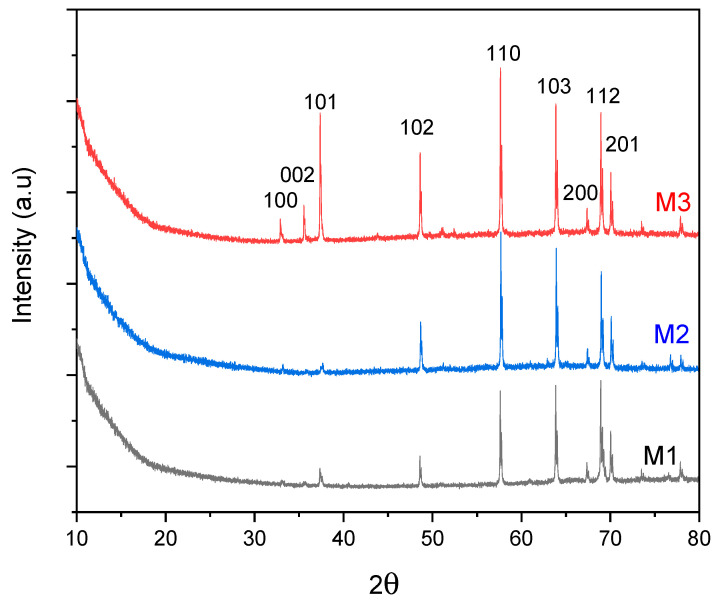
X-ray diffraction (DRX) patterns of M1, M2, and M3 membranes (JCPDS card No. 36-1451).

The XRD patterns of samples M1, M2, and M3 clearly demonstrate the systematic effect of increasing ZnO layer thickness on their crystalline structure (Figure 3). As the ZnO thickness increases, the intensity of the diffraction peaks characteristic of the hexagonal wurtzite phase progressively increases, indicating a larger crystalline volume and enhanced overall crystallinity [25]. The detected reflections correspond to the ZnO crystallographic planes (110), (103), (200), (112), (201), (004), and (202), in good agreement with the standard data reported in JCPDS card No. 36-1451, and are observed for all three membranes.

The similar XRD patterns of M1 and M2 confirm that increasing the ZnO layers from one to two does not change the ZnO crystal structure, but mainly increases the ZnO thickness, resulting in slightly enhanced diffraction intensity. Additionally, diffraction peaks indexed to the (002), (101), and (102) planes, which are of very low intensity in M1 and M2, become distinctly resolved in sample M3. This evolution suggests improved crystallinity and the development of preferential orientations at higher thicknesses. These findings are consistent with previously reported studies, which indicate that thicker ZnO films generally exhibit superior crystalline quality [30].

#### 3.1.2. FTIR Analysis

**Figure 4:**
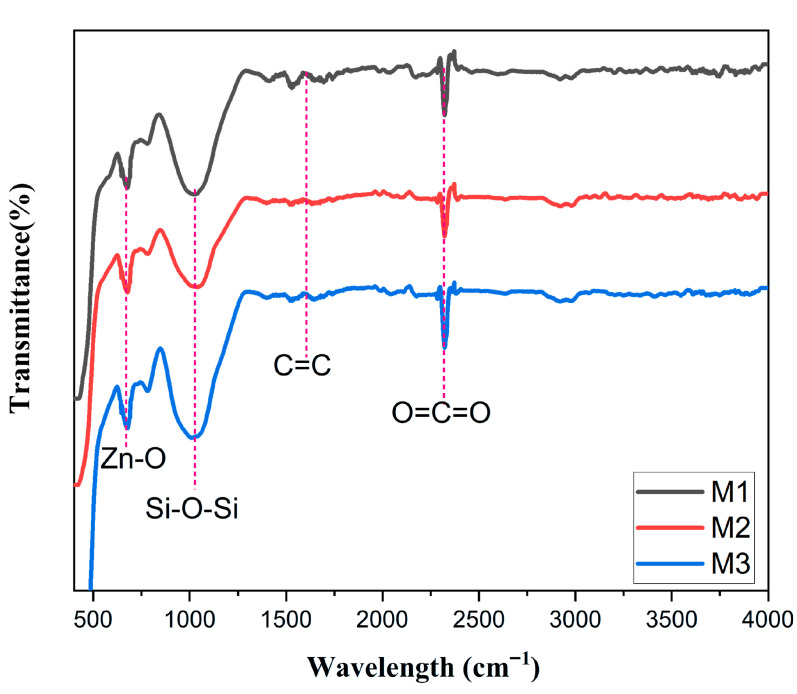
Fourier transform infrared (FTIR) spectra of M1, M2, and M3 membranes.

The FTIR spectra reveal the evolution of absorption bands as the ZnO layer thickness increases in membranes M1, M2, and M3 (Figure 4). A progressive reduction in overall transmittance from M1 to M3 is consistent with the Beer–Lambert law, since thicker layers contain more light-absorbing material and thus attenuate infrared transmission more strongly [39]. In the low-frequency region (400–600 cm^−1^), the characteristic Zn–O vibration band increases in intensity from M1 to M2 and remains strong for M3, indicating a larger volume of crystallized ZnO within the films [40].

At higher frequencies, a broad absorption band between 3200 and 3600 cm^−1^ is observed, which is generally attributed to surface-adsorbed water and hydroxyl groups. The persistence of this band across all membranes, without significant changes in intensity or shape, can be explained by the intrinsic surface chemistry of both ZnO and the ceramic substrate. ZnO is hydrophilic and can readily adsorb moisture from the surrounding environment, leading to the formation of surface hydroxyl groups. In addition, ceramic supports inherently contain surface hydroxyls that remain detectable after ZnO deposition. Therefore, the band observed in this region reflects the presence of adsorbed water and surface hydroxyl groups commonly found in ZnO–ceramic systems [41]. Sharp peaks observed near 2350 cm^−1^ are unrelated to the ZnO film and correspond instead to atmospheric CO_2_ absorption, a common artifact in FTIR spectra when background correction does not fully eliminate ambient contributions [42].

Overall, these spectral variations confirm that increasing ZnO thickness amplifies the material’s characteristic absorption bands and reduces optical transmittance. The results are consistent with the established structural and optical behavior of thin ZnO layers reported in recent studies.

#### 3.1.3. SEM Characterization

**Figure 5:**
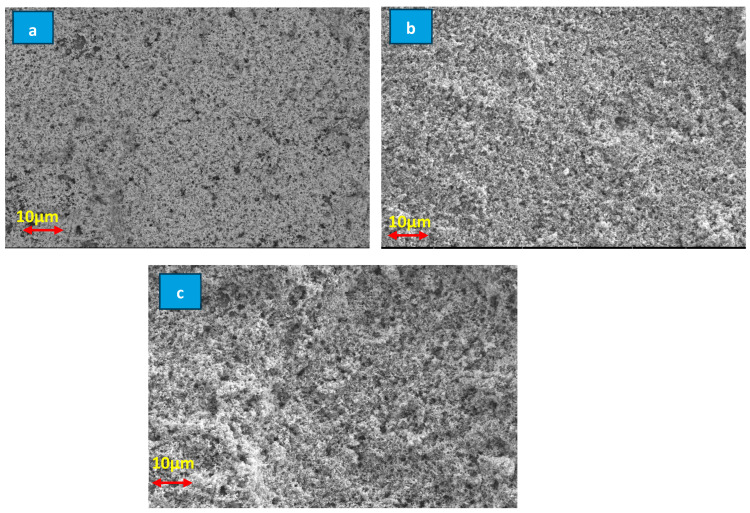
Scanning Electron Microscopy (SEM) images of the membrane morphology of M1 (**a**), M2 (**b**), and M3 (**c**).

Scanning Electron Microscopy (SEM) images reveal a systematic and distinct transformation in the morphology of ZnO as the deposition thickness increases (Figure 5). All three membranes (M1, M2, M3) were fabricated using the same ZnO/PVA coating formulation (4 g ZnO in 100 mL water, mixed 1:1 with 2 wt% PVA), differing only in the number of spin-coated layers. In the initial layer, the film is thin and discontinuous, consisting predominantly of isolated nanoparticles, indicating the early nucleation phase of ZnO. During this stage, crystalline islands form in a dispersed configuration before undergoing lateral expansion, a behavior that aligns well with the existing literature on low-charge deposits or limited growth durations [43,44].

The intermediate layer displays a more homogeneous and well-defined structure, indicative of grain coalescence and the initiation of continuous film formation. The surface density is enhanced, characterized by closely packed particles, which illustrates the “island coalescence” growth mode that is typical of metal oxides [45].

In the third layer, the film is more compact, and exhibits homogeneity, completely covering the underlying membrane. The topography reveals a tightly packed granular structure, resulting from intensified vertical growth and crystallite aggregation behaviours commonly observed in ZnO deposits once the thickness surpasses the coalescence stage [46].

**Figure 6:**
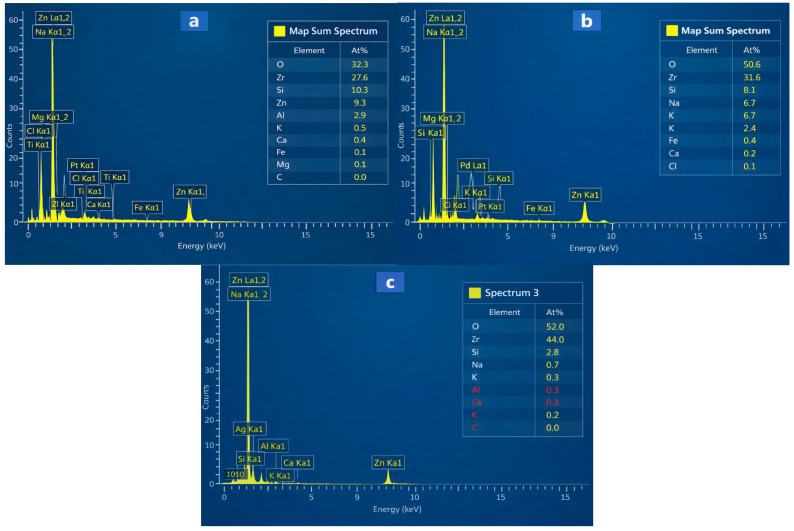
EDX spectra M1 (**a**), M2 (**b**), and M3 (**c**).

The EDX spectra from the image reveal three ZnO-coated ceramic membranes analyzed for photocatalytic wastewater treatment applications (Figure 6) [37]. M1 displays strong Zn and O peaks with subdued substrate signals (Al, Si), indicating a uniform, thick ZnO layer [38,39]. M2 exhibits moderate Zn intensities alongside balanced Al/Si ratios, suggesting partial or thinner coverage from deposition methods such as spin-coating [37]. M3 exhibits lower visual peak heights but a reported 44% Zn (likely), surpassing M1 and M2 due to EDX’s normalized quantification using sensitivity factors [40]. This higher atomic percentage confirms the densest ZnO loading in M3, despite appearances, as at% accounts for matrix effects and overlaps, unlike raw intensities [39]. The green highlights in M3 may emphasize Zn-rich zones, validating superior coating. Overall, M3 offers optimal ZnO content (around 44 at%) for enhanced photocatalysis, followed by M2, then M1, ideal for organic dye degradation on zeolite supports [37]. Uniformity checks via multi-spot analysis are recommended; higher Zn correlates with better reactor performance.

**Figure 7:**
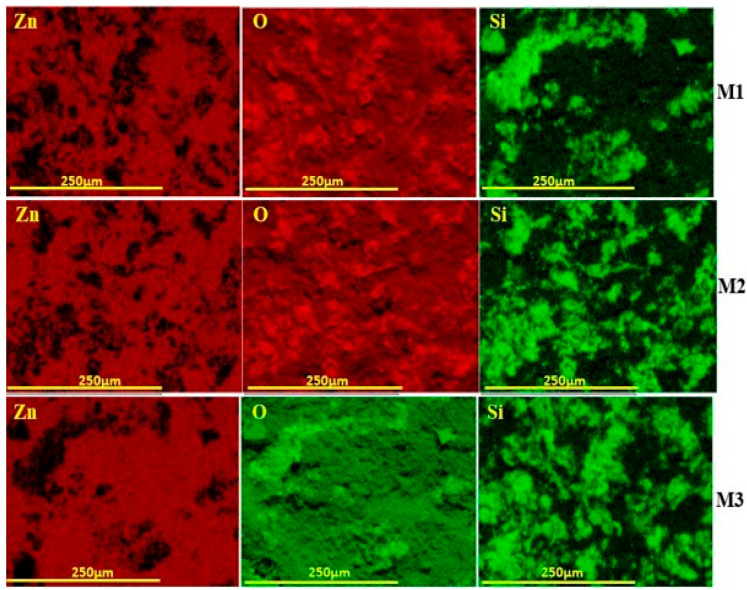
Elemental mapping of M1, M2, and M3 membranes.

Figure 7 shows the elemental mapping of the three membranes, which highlights clear differences in ZnO coverage. M1 shows scattered Zn signals with exposed Si regions, indicating incomplete coating. M2 presents stronger and more evenly distributed Zn and O signals, suggesting better dispersion across the surface. M3 demonstrates the most uniform Zn distribution with minimal Si visibility, confirming continuous and well-adhered ZnO layers. Overall, M3 shows higher ZnO loading, more uniform surface coverage, and enhanced structural features compared to M1 and M2, which suggest a potential for improved photocatalytic performance.

The porosity results followed a clear decreasing trend: the pristine membrane exhibited the highest porosity (39.2%), which progressively declined with increasing ZnO surface coating, reaching 33.6% for the M3 membrane. This reduction is attributed to partial pore coverage by the ZnO layer, which decreases the void volume and increases surface compactness.

#### 3.1.4. Determination of Membrane Permeability

**Figure 8:**
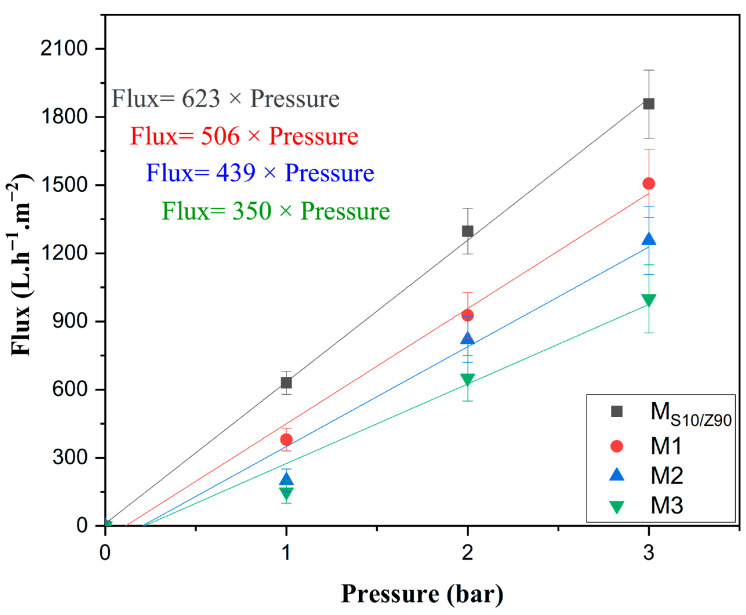
Determination of water permeability for MS10/Z90 support and M1, M2, and M3 coated membranes.

Figure 8 shows pure water permeability for an uncoated ceramic membrane (MS10/Z90) and three membranes coated with varying ZnO layers (M1: 1 layer, M2: 2 layers, M3: 3 layers). Flux decreases as the number of layers increases, with M1 exhibiting the highest permeability across all tested pressures, followed by progressively lower fluxes for M2 and M3. Each dataset follows a straight-line relationship with pressure, indicating reliable pressure-proportional flow governed by membrane resistance [39].

This reduction stems primarily from the added ZnO restricting pore openings and extending flow paths, which outweighs any gains from the material’s water-attracting properties [47,48]. In practice, a single layer (M1) maintains strong throughput suitable for initial filtration stages, while three layers (M3) emphasize longer pollutant exposure for photocatalytic wastewater treatment, even at halved fluxes [40].

Studies on similar ZnO-modified ceramics confirm this pattern, noting that thicker or multi-layer coatings consistently lower water flux due to diminished porosity, though they enhance degradation under light [49]. For your setup, evaluating flux recovery after fouling cycles will clarify if M3’s lower permeability pays off in reduced maintenance for dye removal [48].

### 3.2. Photocatalytic Activity

**Figure 9:**
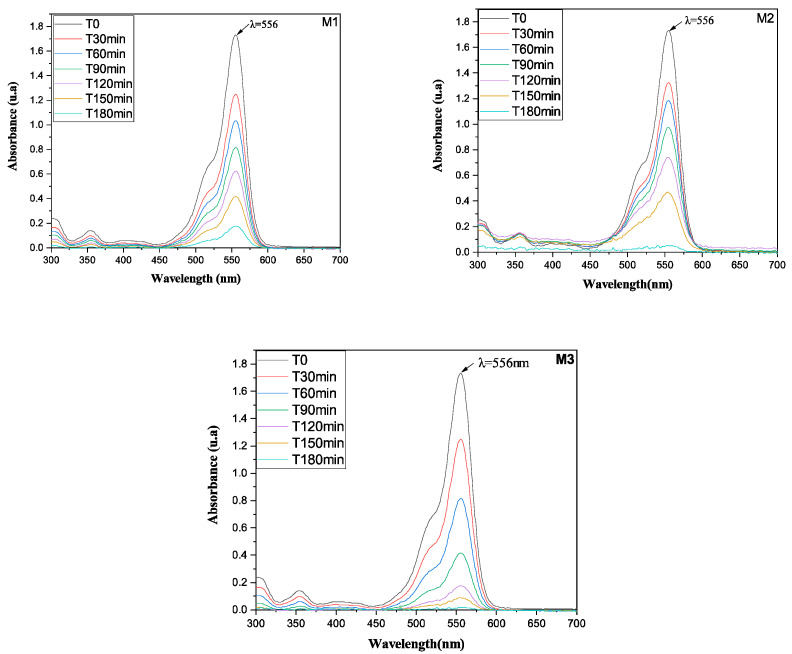
UV–visible absorption spectra showing the decrease in RhB concentration, reflected by the disappearance of the characteristic absorption band at 556 nm during photocatalytic treatment using M1, M2, and M3 under UV irradiation. Initial RhB concentration: 10 mg·L^−1^.

The photocatalytic performance was evaluated by monitoring the decrease in RhB concentration, reflected by the attenuation of its characteristic absorption band at 556 nm [50]. (Figure 9). This decrease corresponds to the disappearance of the chromophore structure, indicating both decolorization and molecular degradation of RhB. Membrane M1 demonstrated the least activity, with only a gradual attenuation of the peak intensity over 180 min, resulting in a high residual dye concentration. Membrane M2 showed moderately improved efficiency, achieving a more substantial reduction in absorbance, though significant dye persistence remained. Membrane M3 exhibited superior photocatalytic performance, characterized by a rapid and nearly complete elimination of the characteristic absorption peak, indicating extensive degradation of the chromophore [51]. Consequently, the spectral analysis establishes a clear efficacy order: M1 < M2 < M3. This hierarchy is quantitatively supported by the comparative rates of absorbance decay and the final degree of dye degradation [52].

### 3.3. Kinetic Study

**Figure 10:**
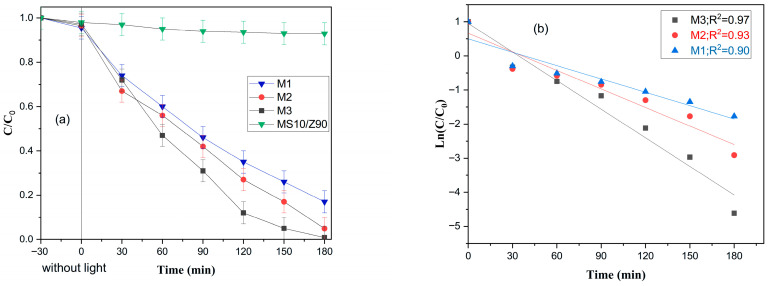
(**a**) Evolution of Photodegradation of RhB by MS 10/Z90, M1, M2, and M3 at concentration = 10 mg·L^−1^ versus time (Conditions: RhB 10 mg·L^−1^; pH = 6.5; T = 25 °C; UV irradiation (λ = 365 nm, 18 W); distance = 3 cm; illuminated area = 23.75 cm^2^; irradiance = 4.2 mW·cm^−2^). (**b**) Ln(C/C_0_) vs. time plot of RhB conversion using M1, M2, and M3 membranes.

Figure 10 depicts the photocatalytic degradation kinetics, monitored over 180 min of UV irradiation, establish a clear performance hierarchy among the membranes [53]. For the bare membrane (MS 10/Z90), RhB removal under UV irradiation is relatively low, resulting from the combined effects of weak adsorption and direct photolysis. The absence of a photocatalyst prevents the generation of reactive species, leading to a much slower degradation rate compared to the ZnO-decorated membrane. For the ZnO-coated membranes, the presence of the photocatalytic layer significantly enhances RhB degradation.

The degradation profiles reveal that membrane M3 achieves the highest RhB removal, followed by M2, and then M1. This is quantitatively supported by the relative C/C_0_ values at the endpoint. For M3, the C/C_0_ value approaches zero, signifying nearly complete degradation of the dye (99.1%). Membrane M2 exhibits a slightly higher residual concentration, indicating strong but comparatively lower activity (94.56%). In contrast, membrane M1 demonstrates the highest C/C_0_ value, confirming it possesses the least effective photocatalytic performance under these conditions (83%).

Overall, the data conclusively ranks the materials. Membrane M3 demonstrates superior efficacy, facilitating near-total RhB degradation [54]. Membrane M2 shows intermediate activity, while M1, despite achieving significant removal over the duration, exhibits the weakest performance. This order of M3 > M2 > M1 is directly derived from the rate and extent of dye decomposition observed in the degradation curves [55].

The plot of Ln(C/C_0_) as a function of irradiation time shows that RhB degradation over the three membranes can be well described by a pseudo-first-order kinetic model [56]. The linear evolution of Ln(C/C_0_) with time and the relatively high correlation coefficients (0.90–0.97) confirm that the Langmuir–Hinshelwood expression reduces to first-order behavior under the experimental conditions used [56].

**Figure 11:**
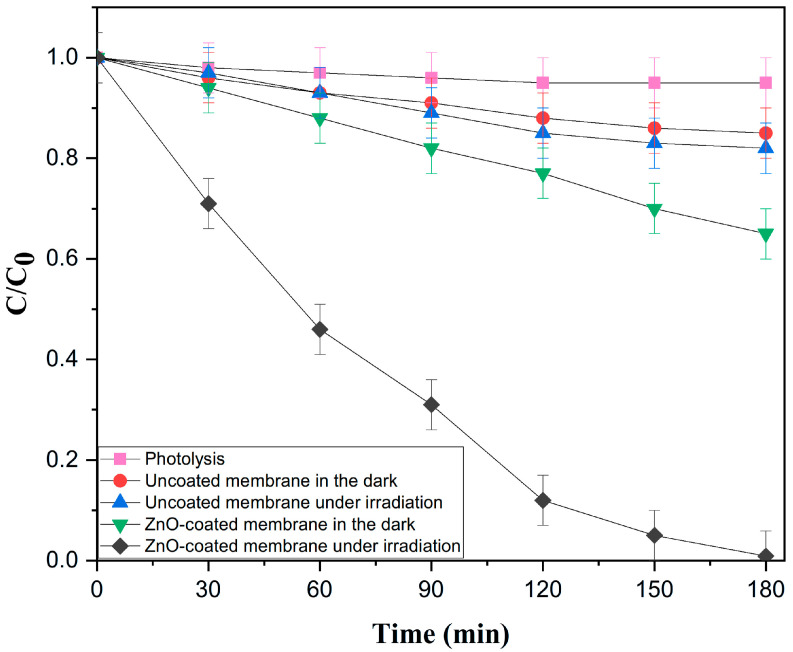
Evolution of RhB photodegradation under control and photocatalytic conditions. Conditions: (Conditions: RhB 10 mg·L^−1^, pH = 6.5; T = 25 °C, UV irradiation (λ = 365 nm, 18 W), distance = 3 cm, illuminated area = 23.75 cm^2^, irradiance = 4.2 mW/cm^2^). Control experiments: light only (direct photolysis), uncoated membrane under irradiation (MS 10/Z90 + UV), ZnO-coated membrane in the dark (adsorption), ZnO-coated membrane under irradiation (photocatalysis).

Figure 11 displays the apparent rate constants, which are found to be 2.8 × 10^−2^, 1.8 × 10^−2^, and 1.3 × 10^−2^ min^−1^ for M3, M2, and M1, respectively, indicating that M3 accelerates the disappearance of RhB much more efficiently than the other two membranes [56]. This trend reflects an enhancement of the photocatalytic properties of M3, which can be attributed to its specific structural and/or compositional features (such as higher density of active sites, better dispersion of the photocatalyst, or more favorable charge separation) compared with M1 and M2. Consequently, the kinetic analysis demonstrates that the photocatalytic performance follows the order M3 > M2 > M1, in agreement with the degradation profiles obtained from the (C/C_0_) versus time curves.

To confirm that RhB removal was due to photocatalytic activity, a full set of control experiments was conducted under the same conditions to separate the effects of direct photolysis, adsorption, and photocatalysis using optimal membrane M3 (Figure 11). Direct photolysis (UV light alone) removed only 5% of RhB after 180 min, showing that the dye is stable under UV irradiation. The uncoated membrane showed minimal removal in the dark (15%) and under UV (23%), confirming it has no photocatalytic activity. For the ZnO-coated M3 membrane, adsorption in the dark accounted for 35% removal, establishing the baseline for dye retention on the surface. Under UV irradiation, the M3 membrane achieved (99.1%) total removal. Although both conditions show dye removal, the mechanisms are fundamentally different. In the dark, removal is due solely to reversible adsorption, while under UV, photogenerated reactive species continuously degrade the adsorbed dye, freeing active sites for further reaction. These controls confirm that the enhanced performance of the M3 membrane results from true photocatalytic degradation, not from adsorption or photolysis alone.

### 3.4. Photocatalytic Regeneration and Permeability Recovery

Cleanability is a key requirement for photocatalytic membranes, as it directly affects their long-term performance, fouling resistance, and operational stability. In this context, the cleanability behavior and structural durability of the ZnO-coated membrane (M3) were evaluated after photocatalytic treatment under UV irradiation using XRD, FTIR, and SEM analyses.

**Figure 12:**
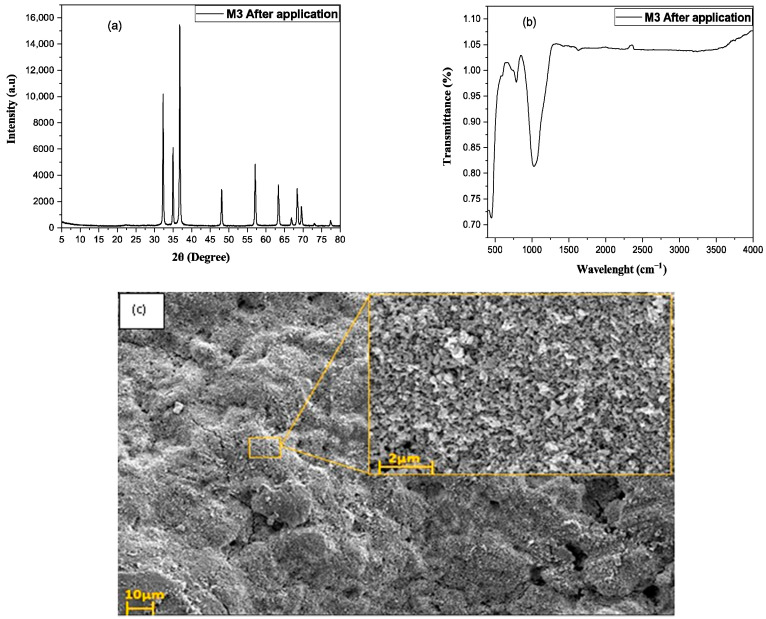
X-ray diffraction (XRD) pattern of M3 after application (**a**), Fourier transform infrared (FTIR) spectra of M3 after application (**b**), analysis of the bottom surface of the fouled membrane (**c**).

XRD results (Figure 12a) show that the diffraction peaks remain at the characteristic 2θ positions of zeolite and wurtzite ZnO, including ZnO peaks around 31–37°. This indicates that the crystal phases are stable and unaffected by the photocatalytic process [20]. No noticeable shifts or disappearance of peaks are observed after use. The similar peak shapes and intensities before and after testing suggest that there is no significant loss of ZnO. Overall, the M3 coating exhibits good structural and mechanical stability during cleanability cycles [57].

FTIR analysis after photocatalysis shows a strong reduction or disappearance of bands related to organic contaminants (Figure 12b), such as C-H stretching vibrations at 2900–3000 cm^−1^ and C=O or C-O bands in the 1700–1100 cm^−1^ region. At the same time, the characteristic framework vibrations of zeolite (Si-O-Si and Al-O-Si below 1100 cm^−1^) and the Zn-O bands remain unchanged [58]. This confirms that the inorganic structure is preserved during photocatalytic treatment. An increase in the broad O-H stretching band around 3200–3600 cm^−1^ is also observed. Together, these changes indicate effective degradation of organic foulants and regeneration of a clean, hydrophilic surface [59].

The SEM image of the ZnO-coated membrane (M3) after UV irradiation shows a bottom surface that appears fully regenerated (Figure 12c), indicating that the cleaning step was highly effective. The surface is uniformly exposed, with a continuous granular texture characteristic of the underlying support and ZnO layer, and no distinct cake layer, clusters, or deposits attributable to RhB or other foulants can be discerned [21]. This morphology suggests that photocatalytically generated oxidizing species have removed the adsorbed dye and associated organic matter from the membrane interface, leaving the pores and surface features free of observable pollutants [60].

In addition, the regeneration capacity was evaluated by measuring water permeability after photocatalytic treatment over three consecutive cycles (Table 4). As shown in the table below, the permeability after cleaning remained consistently high, with permeability recovery ratios (PRR) of 97.7%, 98.0%, and 97.9% for cycles 1, 2, and 3, respectively. These results confirm effective in situ regeneration of the membrane surface after each cycle (Table 4). While the absolute permeability showed a slight gradual decline from 350 to 328 L·h^−1^·m^−2^·bar^−1^ over the three cycles, likely due to residual irreversible fouling or minor structural changes, the consistently high PRR values demonstrate excellent repeatability and durability of the cleanability function. These findings provide strong evidence of the membrane’s potential for sustained long-term operation.

### 3.5. Rhodamine B Degradation Mechanism Using M3 Membrane

The photocatalytic activity of the ZnO coating, immobilized on the Zeolite-Smectite membrane, was evaluated through the degradation of Rhodamine B (RhB) under UV irradiation **(365 nm, 18 W**). ZnO is a wide-band-gap semiconductor with a band gap of approximately 3.20–3.37 eV that absorbs primarily in the UV region, with conduction band (CB) and valence band (VB) positions at −0.6 eV and 2.7 eV, respectively [36]. Upon UV irradiation, the ZnO thin film undergoes photoexcitation, generating electron–hole pairs (Equation (6)).

The subsequent chemical mechanism involves the transformation of these charge carriers into reactive oxygen species (ROS). The photogenerated holes (h^+^) in the valence band can oxidize surface-adsorbed water molecules or hydroxide ions to produce highly reactive hydroxyl radicals (•OH) (Equation (7)). Concurrently, the electrons (e^−^) in the conduction band reduce dissolved oxygen to form superoxide radicals (O_2_•^−^) (Equation (8)). These ROS are responsible for the oxidative degradation of RhB adsorbed on the membrane surface, proceeding through N-deethylation, chromophore cleavage, and partial aromatic ring opening, leading to progressive dye degradation and decolorization [61,62].

(7) $$ZnO+h\nu \to \left(e^{-}\right.+\left.h^{+}\right)$$

(8) $$h^{+}+H_{2}O\to H+{OH}^{•}$$

(9) $$h^{+}+{OH}^{-}\to {OH}^{•}$$

(10) $$e^{-}+O_{2}\to O_{2}^{•.-}$$

(11) $$O_{2}^{•-}+H_{2}O\to {2OH}_{2}^{•}$$

(12) $${OH}^{•}+Organic\;pollutants\to {CO}_{2}+H_{2}O$$

**Figure 13:**
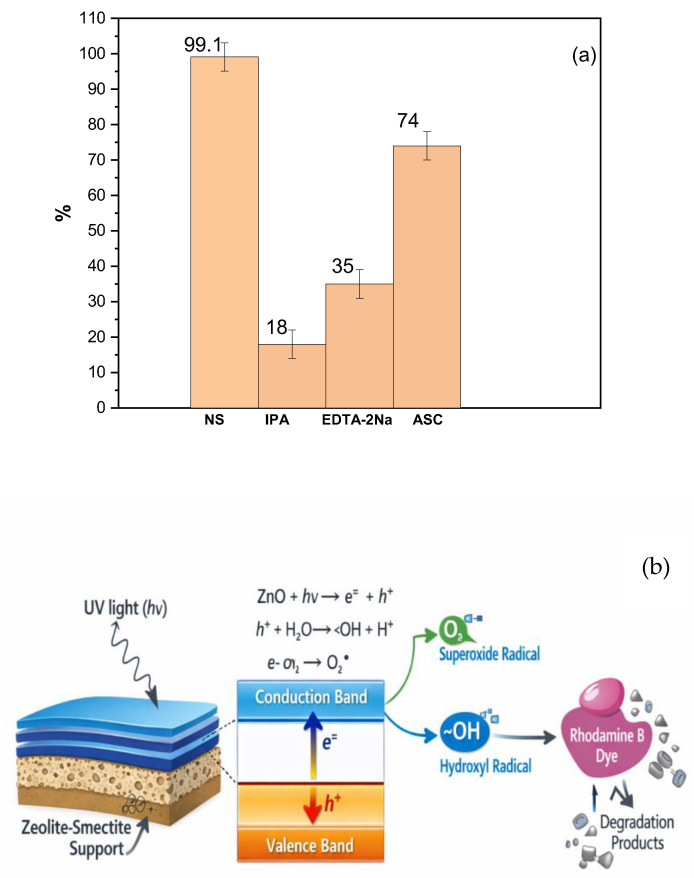
Effect of various scavengers on the degradation efficiency of RhB over supported ZnO. Experimental conditions: M3 (3 layers), [RhB] = 10 mg·L^−1^, reaction time = 180 min. The UV source (λ = 365 nm, 18 W) was positioned at a distance of 3 cm from the membrane surface, providing an irradiance of approximately 4.2 mW·cm^−2^ measuredusing a radiometer. Scavenger concentrations: 0.5 mM. The control experiment (No Scavenger) represents the baseline degradation efficiency (**a**), proposed mechanism of RhB photodegradation, and surface regeneration using the M3 membrane. Insitu generation of reactive oxygen species facilitates degradation of organic foulants, contributing to flux recovery and operational stability (**b**).

To elucidate the dominant degradation pathways and validate the proposed mechanism, radical scavenging experiments were performed. Specific scavengers—isopropanol (IPA) for •OH, ethylenediaminetetraacetic acid disodium salt (EDTA-2Na) for photogenerated holes (h^+^), and ascorbic acid (ASC) for superoxide radicals (O_2_•^−^) were introduced to isolate the contribution of each ROS [61,62]. As shown in Figure 13a, the introduction of these scavengers resulted in a pronounced suppression of RhB degradation, confirming the involvement of multiple oxidative species.

The pristine ZnO catalyst under UV irradiation achieved nearly complete RhB removal (>99%). However, degradation efficiencies decreased markedly to approximately 18%, 35%, and 74% in the presence of IPA, EDTA-2Na, and ASC, respectively. The strong inhibition observed with IPA and EDTA-2Na confirms that hydroxyl radicals and direct hole oxidation are the primary degradation pathways. The comparatively weaker effect of ASC suggests that superoxide radicals play a secondary role in this system, a finding that is consistent with prior reports on ZnO-based photocatalysts [35,36]. Furthermore, the ceramic support itself has been shown to contribute to the overall photocatalytic performance [63].

In summary, the scavenger assays demonstrate that RhB photodegradation over the ZnO ceramic composite proceeds predominantly via hydroxyl radicals and direct hole oxidation, with superoxide radicals providing a minor contribution. Figure 13b indicates that simultaneously, the continuous in situ generation of oxidative radicals can contribute to the degradation of organic foulants deposited on the membrane surface, improving membrane cleanability during operation. At the same time, the enhanced hydrophilicity associated with the ZnO coating promotes the formation of a stable hydration layer, collectively imparting a cleanability function and mitigating membrane fouling during operation. On the whole, the photocatalytic membrane provides a clear practical advantage, as it can be readily separated from the treated water, effectively avoiding secondary pollution and reducing additional post-treatment costs.

## 4. Comparative Study

Table 5 provides a comparative evaluation of membranes, hybrid processes, photo-catalysts, and nanoparticles for the removal of Rhodamine B dye. A detailed analysis of Rhodamine B (RhB) removal efficiency across different ZnO-based membrane systems highlights the critical role of structural design, support material, and operating conditions in determining performance. For instance, Bentonite-coated perlite membranes achieved 80.1% removal at 50 ppm RhB, with moderate flux values under 4bar pressure [64]. In contrast, hybrid systems combining ZnO photocatalysts with ceramic nanoporous membranes demonstrated superior performance, reaching 96% removal at a much higher dye concentration (500 ppm), accompanied by fluxes between 124.8 and 85.8 L·h^−1^·m^−2^ [65]. On the other hand, ZnO-layered PTFE membranes exhibited variable efficiencies ranging from 42% to 97%, depending on dye type and [66]. Likewise, photocatalytic composites such as ZnOCu_0.5_O heterostructures achieved 73% removal at 10 ppm RB [33], while In-doped ZnO nanoparticles under UV irradiation showed comparable efficiency (73%) at 20 ppm RhB [67]. ZnO-layered porous SiC supports provided consistently high removal (91–95%) with excellent flux values exceeding 250 LMH at low operating pressure [30]. In this work, the ZnO-layered porous MS10/Z90 support developed achieved the highest efficiency, with 99.1% removal at 10 ppm RhB, confirming the effectiveness of the structured ZnO layer in enhancing photocatalytic degradation and membrane performance. Overall, this comparison demonstrates that ZnO-based membranes consistently outperform conventional supports, with removal efficiency strongly influenced by membrane architecture, dopants, and operating conditions. The present study establishes the MS10/Z90-supported ZnO membrane as a highly effective system for RhB removal, aligning with and surpassing reported performances in the literature.

## 5. Conclusions

This work demonstrates the successful fabrication of a low-cost ceramic photocatalytic membrane by depositing ZnO onto a zeolite/smectite support using the spin-coating technique. The coating suspension used for all membranes consisted of 4 wt% ZnO, obtained by mixing a ZnO dispersion (4 g in 100 mL water) with an equal volume of a 2 wt% PVA solution. Optimization of the active layer revealed that membranes coated with two (M2) and three (M3) ZnO layers exhibited homogeneous, compact, and defect-free morphologies, as confirmed by SEM analysis, with M3 showing superior structural uniformity.

The M3 membrane, containing 4 wt% ZnO, achieved a Rhodamine B degradation efficiency of 99.1% under UV irradiation after 180 min, as determined by UV–vis spectrophotometry through the decrease in absorbance at 556 nm, which corresponds to the disappearance of the characteristic chromophore band and reflects both decolorization and degradation of the dye. The degradation kinetics followed a pseudo-first-order model, with the apparent rate constant increasing with the number of ZnO layers. This enhanced performance is attributed to the higher density of active sites provided by the thicker catalyst layer, which facilitates greater generation of reactive oxygen species. Although the addition of ZnO layers resulted in a progressive reduction in water permeability, the trade-off between permeability and catalytic activity was considered acceptable given the substantial improvement in pollutant degradation.

Reactive species scavenging assays identified hydroxyl radicals (•OH) as the primary oxidizing species responsible for RhB conversion. Following photocatalytic operation, water permeability measurements demonstrated an almost complete flux recovery, achieving a permeability recovery ratio (PRR) of 97.7% after three cleaning cycles. This observation suggests that the ZnO layer enables in situ photocatalytic degradation of organic foulants, contributing to membrane regeneration. Structural analyses, including XRD and SEM, confirmed the preservation of the ZnO wurtzite phase and the integrity of the ceramic support after operation.

Despite these promising results, this study has certain limitations that should be acknowledged. The evaluation of cleanability was conducted over a limited number of regeneration cycles (three), which, while demonstrating repeatability, does not fully capture long-term operational stability or the potential for gradual performance decline. Additionally, the experiments were performed using a single model pollutant (Rhodamine B) in deionized water, without the complexity of real industrial wastewater matrices that contain competing organic matter, salts, and variable pH conditions that may influence photocatalytic efficiency and fouling behavior.

Therefore, future research should systematically evaluate performance over multiple sequential fouling/regeneration cycles, quantifying flux recovery, irreversible fouling, and repeatability to validate the long-term durability of the photocatalytic cleanability function. Validation using complex industrial effluents and long-term stability studies under continuous operation are essential to assess practical viability. Finally, scalable fabrication and integration into modular systems are critical for enabling sustainable, energy-efficient wastewater remediation.

## Figures and Tables

**Table 1:** The characteristics of RhB.

Dye	Molecular Formula	Molecular Weight (g·mol^−1^)	Wavelength (λ_max_) (nm)	Charge	Chemical Structure
RhB	C_28_H_31_N_2_O_3_Cl	479.01	556	Cationic	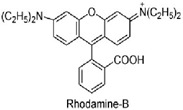

**Table 2:** Ceramic support characteristics; **Data adapted from reference** [28].

Parameters	Values
Water permeability (L·h^−1^·m^−2^·bar^−1^)	623
Mechanical strength (MPa)	23
Average pore size (µm)	0.98
Permeability (%)	39.2

**Table 3:** Membrane samples and the corresponding number of ZnO spin-coated layers.

Membrane Code	M1	M2	M3
Number of coating layers	1	2	3

**Table 4:** Permeability recovery of the M3 membrane over three photocatalytic cleaning cycles.

Cycle	Initial Permeability (L·h^−1^·m^−2^·bar^−1^)	Permeability After Degradation (L·h^−1^·m^−2^·bar^−1^)	Permeability After Cleaning (L·h^−1^·m^−2^·bar^−1^)	PRR (%)
1	350	305	342	97.7
2	342	298	335	98.0
3	335	292	328	97.9

**Table 5:** Comparison of Rhodamine B Removal Efficiency Using ZnO-StructuredMembranes.

Structured Membrane	Operational Conditions	Performances	Reference
Bentonite-coated perlite membrane	Initial concentration of RhB = 50 ppm Filtration under 4 bar	Dye removal Efficiency = 80.1% Permeability = 21 L·h^−1^·m^−2^·bar^−1^	[64]
ZnO photocatalyst coupled with ceramic Nanoporous membrane (hybrid system)	Initial concentration of RhB = 500 ppm; pH = 7 Operation time = 90 min	Dye removal Efficiency = 96% Water flux Permeability = 31–21 L·h^−1^·m^−2^·bar^−1^	[65]
ZnO layered polytetrafluorethylene membrane (Atomic layer deposition)	Initial concentration of RhB = 10–100 ppm Time of operation = 240 min	Removal Efficiency = 42–97%	[32]
ZnO-Cu_0.5_O hetero composite structure (photocatalyst)	Initial concentration of RhB = 10 ppm Photocatalyst dose = 0.05 g Visible light from a 400 W (metal halide lamp) Operation time = 120 min	Removal Efficiency = 73%	[33]
6% In-doped ZnO nanoparticles (under UV radiation) RhB	Initial concentration of RhB = 20 ppm Operation time = 120 min pH = 6.2 The UV irradiation was carried out by using a 125 W (311 nm) medium-pressure Hg arc lamp	Removal Efficiency = 76%	[67]
ZnO layered porous SiC support	Initial concentration of RhB = 4–50 ppm pH = 3, 7, and 11 Filtration pressure = 0.5 bar Operation time = 50 min	Removal Efficiency = 91–95% Permeability = 306–205 L·h^−1^·m^−2^·bar^−1^	[30]
ZnO layered porous support MS10/Z90	Initial concentration of RhB = 10 ppm; pH = 6.5 Photocatalysis for 180 min	Removal Efficiency = 99.1%	This work

## Data Availability

The data relevant to this work are presented in the manuscript.
